# Accuracy of Xpert MTB/RIF assay for the diagnosis of tuberculous pleural effusion

**DOI:** 10.1002/jcla.24185

**Published:** 2021-12-17

**Authors:** Yi‐Ran Qiu, Yu‐Yan Chen, Xin‐Ran Wu, Ya‐Ping Li, Xun‐Jie Cao, Zi‐Yuan Yu, Min Lin, Qiu‐Yin Li, Ji‐Chun Chen, Xin Yin, Shu‐Chang Weng, Xu‐Guang Guo

**Affiliations:** ^1^ Department of Clinical Laboratory Medicine The Third Affiliated Hospital of Guangzhou Medical University Guangzhou China; ^2^ Department of Clinical Medicine The Second Clinical School of Guangzhou Medical University Guangzhou China; ^3^ Department of Clinical Medicine The Fifth Clinical School of Guangzhou Medical University Guangzhou China; ^4^ Department of Clinical Medicine The Third Clinical School of Guangzhou Medical University Guangzhou China; ^5^ Department of Chinese and Western Clinical Medicine The Chinese and Western Clinical School of Guangzhou Medical University Guangzhou China; ^6^ Department of Pediatrics The pediatrics school of Guangzhou Medical University Guangzhou China; ^7^ Key Laboratory for Major Obstetric Diseases of Guangdong Province The Third Affiliated Hospital of Guangzhou Medical University Guangzhou China; ^8^ Key Laboratory of Reproduction and Genetics of Guangdong Higher Education Institutes The Third Affiliated Hospital of Guangzhou Medical University Guangzhou China

**Keywords:** diagnostic, mycobacterium tuberculosis, pleural effusion, tuberculosis, Xpert MTB/RIF

## Abstract

**Background:**

Tuberculosis poses a severe threat to human health. At present, compared with the traditional diagnostic methods for tuberculosis pleural effusion, such as Löwenstein–Jensen culture, pleural biopsy, and Ziehl–Neelsen smear microscopy, Xpert MTB/RIF was regarded as an emerging technology for its efficiency. The Xpert MTB/RIF accuracy for tuberculous pleural effusion diagnosis was evaluated in this systematic study.

**Materials and methods:**

We searched the relevant literature published before January 2021 in PubMed, Cochrane, EMBASE, and Web of Science databases. Utilizing Review Manager 5.3 software, the quality of the included literature was evaluated based on the Quality Assessment of Diagnostic Accuracy Studies criteria. Sensitivity, specificity, and the summary receiver operating characteristic curves were plotted and analyzed with Metadisc 1.40 software. We used Stata 12.0 software to evaluate the publication bias of this study.

**Results:**

Eighteen articles were identified in total. The sensitivity of Xpert MTB/RIF in the pleural effusion was 0.24, and specificity was 1.00, respectively. The area under the summary receiver operating characteristic curve was 0.9737, which indicated that the overall accuracy of the Xpert MTB/RIF was high. In addition, based on the Deeks funnel plot, no publication bias of the study was found.

**Conclusion:**

Xpert MTB/RIF is a rapid method with high specificity but relatively low sensitivity for detecting Mycobacterium tuberculosis in pleural effusion. Its less sensitivity made it difficult to be used clinically, but the high specificity suggests that it can be used as a specific diagnostic method for tuberculous pleural effusion.

## Graphical abstract

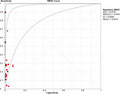

## 1 INTRODUCTION

Given rises to Mycobacterium tuberculosis (MTB), tuberculosis (TB), a chronic disease, has a huge impact on global public health. At the same time, tuberculosis is the primary cause of death of a single infectious agent. Known as the most important and common pathogen in humans, Mycobacterium tuberculosis is the etiological agent of tuberculosis (TB). It can invade various organs throughout the body. Mycobacterium tuberculosis can enter the bloodstream and spread interior or exterior of the lung, which presenting as tuberculosis or extrapulmonary tuberculosis, respectively. According to the World Health Organization (WHO), in 2019, the number of patients newly diagnosed with TB was nearly 10.0 million worldwide, and the annual number of deaths was over 1.4 million.^1^ Tuberculous pleurisy is a common form of extrapulmonary tuberculosis and the principal cause of pleural effusion.^2^

For most people, TB is curable if diagnosed and treated correctly in time. Early detection of TB is the key to the early treatment of tuberculosis (TB). In this way, we can reduce TB‐related morbidity and mortality, as well as transmission. Currently, the traditional diagnostic methods for TB pleural effusion include Löwenstein–Jensen (LJ) culture, pleural biopsy, and Ziehl–Neelsen (ZN) smear microscopy.^3^, ^4^ However, the diagnosis is challenging due to the paucibacillary nature of pleural tuberculosis^5^ and the non‐uniform circulation of MTB. Traditional methods fail to meet expectations in terms of sensitivity, diagnostic time, and the requirements for technicians and instruments, which are difficult to achieve.

Xpert MTB/RIF assay is a rapid, automated PCR test endorsed by WHO for TB. It is a box‐based nucleic acid amplification method, which merely takes a very short time in detecting the Mycobacterium tuberculosis. What's more, Xpert MTB/RIF can detect both MTB and rifampicin resistance in respiratory specimens simultaneously.^6^, ^7^, ^8^ Rifampicin is a crucial drug for the treatment of patients who suffer from tuberculosis. Xpert MTB/RIF assay has the advantages of high sensitivity, specificity, simple operation, low contamination risk, and short turnaround time.^9^, ^10^ This meta‐analysis evaluates the accuracy of Xpert in detecting tuberculous pleural effusion by systematically reviewing all relevant articles.

## 2 MATERIALS AND METHODS

### 2.1 Search strategy and source

Using “Xpert MTB/RIF,” “Tuberculosis pleural effusion,” and their synonyms as the keywords, we conducted a systematic search. According to inclusion criteria, relevant articles published before January 2021 were comprehensively retrieved from four databases, including Web of Science, PubMed, EMBASE, and Cochrane Library.

### 2.2 Inclusion criteria and exclusion criteria

Three researchers screened the retrieved literature in accordance with pre‐defined inclusion and exclusion criteria. Each paper was proofread by two researchers independently. In the situation of disagreement, the third researcher would make the judgment and obtain the complete screening result finally.

The inclusion criteria were summarized as follows: (1) analysis of human specimens, (2) English version, (3) Xpert MTB/RIF was in comparison with another reference standard to test the accuracy of diagnosis for tuberculous pleural effusion, and (4) the data in the article are enough to create a four‐cell table.

The exclusion criteria were summarized as follows: (1) non‐human samples; (2) repeated publications, conference abstracts, letters, case reports, editorials, reviews, and meta‐analyses; (3) lack of four‐grid table data; and (4) the literature lacks a gold standard or Xpert MTB/RIF analysis.

Detailed flowcharts for inclusion and exclusion are shown in additional materials.

### 2.3 Data collection

Data extraction and quality assessment of all literature were first completed independently by two researchers. Results were reviewed, and inconsistencies were discussed by the two researchers. If an agreement cannot be reached, the third researcher will make an evaluation. Finally, a consensus will be reached based on the judgments of the three researchers, summarizing all the results.

### 2.4 Data extraction

Three researchers extracted relevant data of the study articles, including the name of the first author, study design, country, year of publication, sample size, reference standard, and false positive (FP), true positive (TP), true negative (TN), and false negative (FN). After that, three researchers focused on the final extraction results and set up a feature table for the extracted data.

### 2.5 Quality assessment standard

The Quality Assessment for Diagnostic Accuracy Studies (QUADAS‐2)^11^ was used as a criterion to evaluate the quality of the included studies. Afterward, Review Manager (Version 5.3) software was applied to evaluate the diagnostic accuracy of Xpert MTB/RIF. The risk of bias for each study was evaluated using “yes,” “unclear,” and “no,” according to the eleven criteria in the four parts of QUADAS‐2 (patient selection, index test, standard gold method, flow, and time). Charting with the software, we analyzed the risk of bias and suitability issues, including patient selection, indicator trials, reference criteria, procedures, and timing.

### 2.6 Statistical analysis

Sensitivity, specificity, positive‐likelihood ratio (PLR), negative‐likelihood ratio (NLR), and diagnostic odds ratio (DOR) were generated using Meta disc (version 1.40). We plotted and analyzed the summarized receiver operating characteristic (SROC) curves and calculated the area under the curve (AUC). Stata (version 12.0) software was used to draw Deeks funnel plots to assess whether there was bias in the literature. Finally, quality assessment was studied using the Review Manager (version 5.3) software.

## 3 RESULTS

### 3.1 Search results

From the databases mentioned above, 125 relevant articles were identified, including 44 in PubMed, 7 in Cochrane Library, and 74 in the Web of Science. Of the 125 references, 51 were duplicates. A total of 28 articles remained after reviewing the initial selection of titles and abstracts. Then, a further ten articles were excluded after the full‐text screening of the remaining literature for the following reasons: 1 article was a meta‐analysis, two lacked reference standard, five were unable to extract complete data, one was a non‐English article, and another could not be found its full text.

Finally, 18 articles were included for meta‐analysis.^5^, ^12^, ^13^, ^14^, ^15^, ^16^, ^17^, ^18^, ^19^, ^20^, ^21^, ^22^, ^23^, ^24^, ^25^, ^26^, ^27^, ^28^

### 3.2 Characteristics of eligible studies

Data were extracted from the final 18 articles, and feature information, such as the author name and year of publication, is summarized in Table 1.

**TABLE 1:** Specific content of the selected studies (*n* = 18)

No.	First author	Year	Country	Design	Source of specimens	Gold standard	Result			
							TP	FP	TN	FN
1	Friedrich^12^	2011	South Africa	prospective	25	Culture	5	0	5	15
2	Moure^13^	2012	Spain	prospective	31	DNA probes	7	0	5	19
3	Christopher^14^	2013	India	prospective	91	CRS‐1	4	0	66	21
4						CRS‐2	4	0	61	26
5	Porcel^15^	2013	Spain	prospective	67	Auramine stain/Culture/Tissue/ADA	5	0	34	28
6	Lusiba^16^	2014	Uganda	prospective	116	Culture/Histopathology	25	1	28	62
7	Meldau^17^	2014	South Africa	prospective	93	Culture	9	1	52	31
8	Trajman^18^	2014	Brazil	prospective	85	AFB/culture/biopsy	2	0	26	57
9	Coleman^19^	2015	Malawi	prospective	31	Culture	9	0	18	4
10	Rufai^20^	2015	India	prospective	161	Culture	23	0	119	19
11	Che^21^	2017	China	prospective	78	Pathological examination	12	0	18	48
12	Saeed^22^	2017	Pakistan	prospective	158	Culture	30	0	125	3
13	Christopher^5^	2018	India	retrospective	65	CRS	4	0	36	25
14	Sharma^23^	2018	India	prospective	78	CRS	16	0	30	32
15	Galal El‐Din^24^	2019	Egypt	prospective	58	CRS	1	0	12	45
16	Liang^25^	2019	China	retrospective	219	CRS	22	0	64	133
17	Meldau^26^	2019	South Africa	Prospective	133	CRS	14	1	83	35
18	Han^27^	2020	China	prospective	265	Culture	61	0	42	162
19	Sumalani^28^	2020	Pakistan	prospective	148	Microbiologic tests/Clinical diagnosis	9	0	64	75

Abbreviations: ADA, adenosine deaminase; AFB, acid‐fast bacillus; CRS, composite reference standard; FN, false negative; FP, false positive; TN, true negative; TP, true positive.

### 3.3 Quality assessment

**FIGURE 1:**
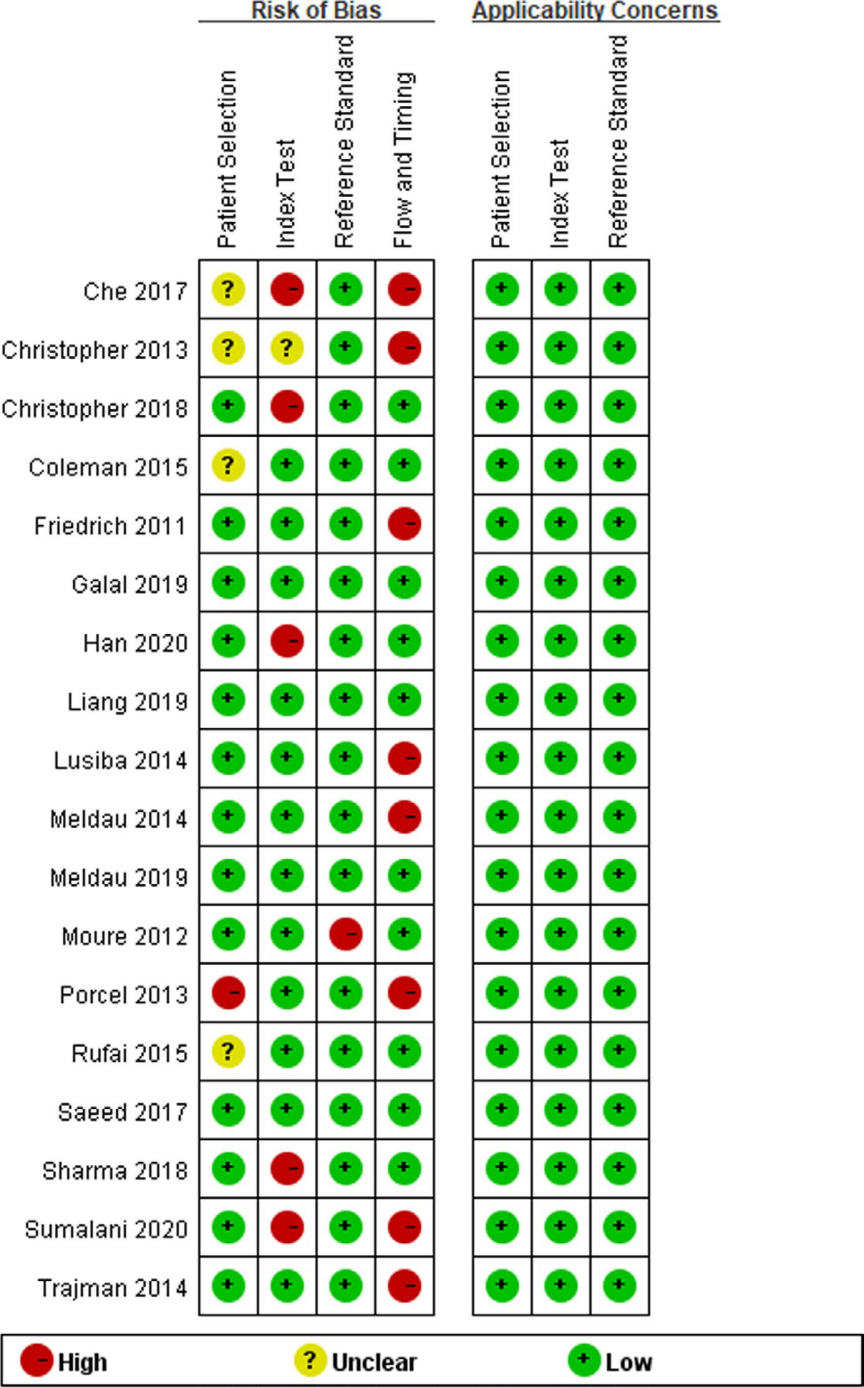
Quality assessment of the selected studies

**FIGURE 2:**
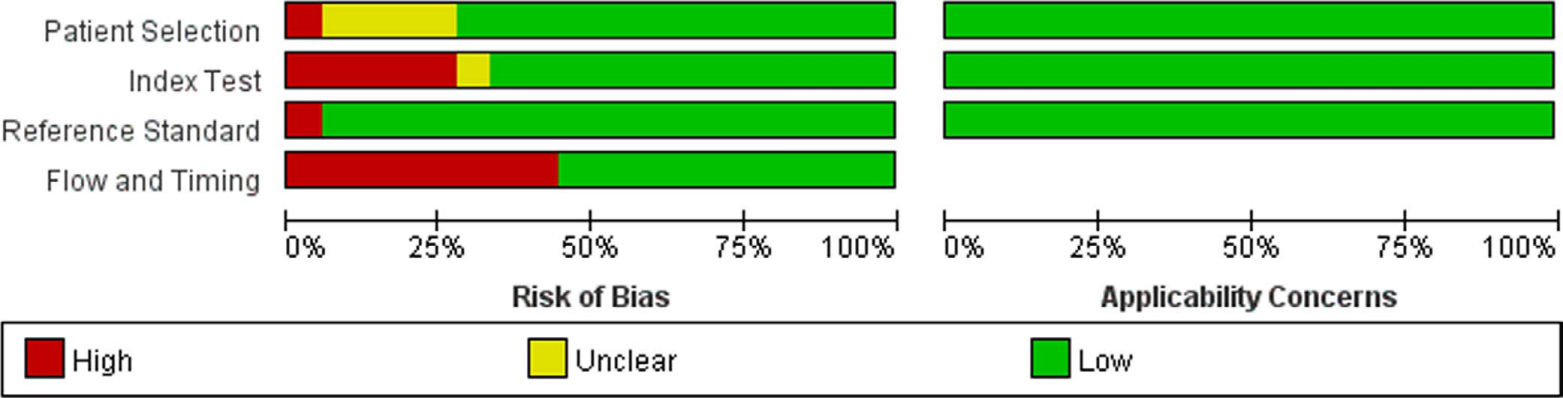
The bias risk and applicability diagram of Xpert for tuberculous pleural effusion

The quality of the 18 articles (Figures 1 and 2) was assessed using QUADAS‐2 as a uniform standard. The results suggested that four articles (22.22%) had an unclear risk of bias in patient selection, while 1 article (5.55%) had a high risk, and the others had a low risk. In terms of index test and reference standard, five articles (27.78%) were judged to be a high risk of bias, 1 article (5.55%) was at unclear risk of bias in index test, and only 1 article (5.55%) was judged to be in a high risk of bias in the reference standard. In the analysis of the patient flow and timing, eight articles (44.44%) were rated as a high risk of bias, while the others had a low risk of bias. Moreover, the applicability concerns of 18 articles indicated low concerns in patient selection, index tests, and reference standards.

### 3.4 Data analysis

MetaDiSc was applied to analyze the fourfold table data from these 18 articles.

**FIGURE 3:**
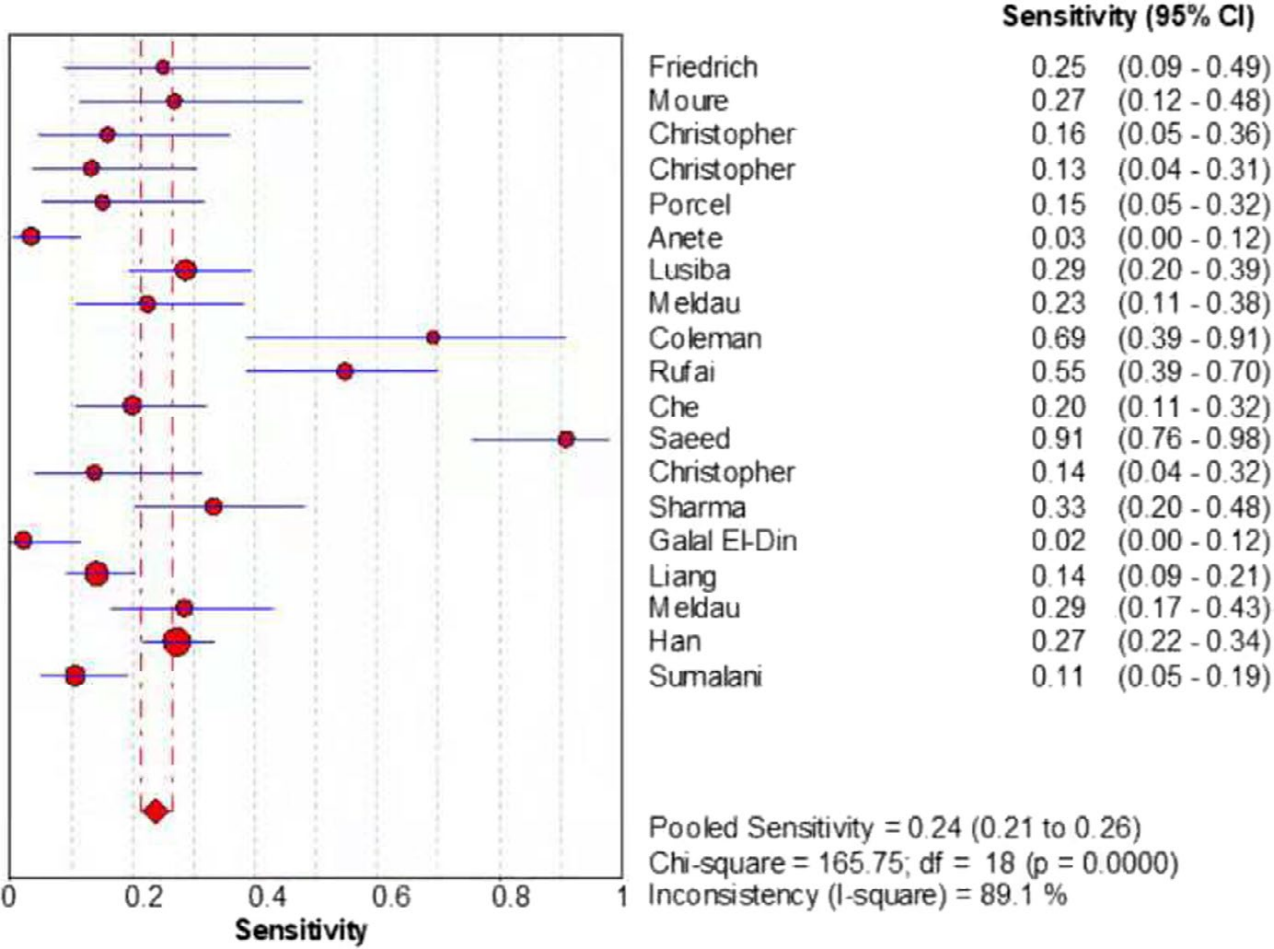
Forest plot of the sensitivity of the included studies

**FIGURE 4:**
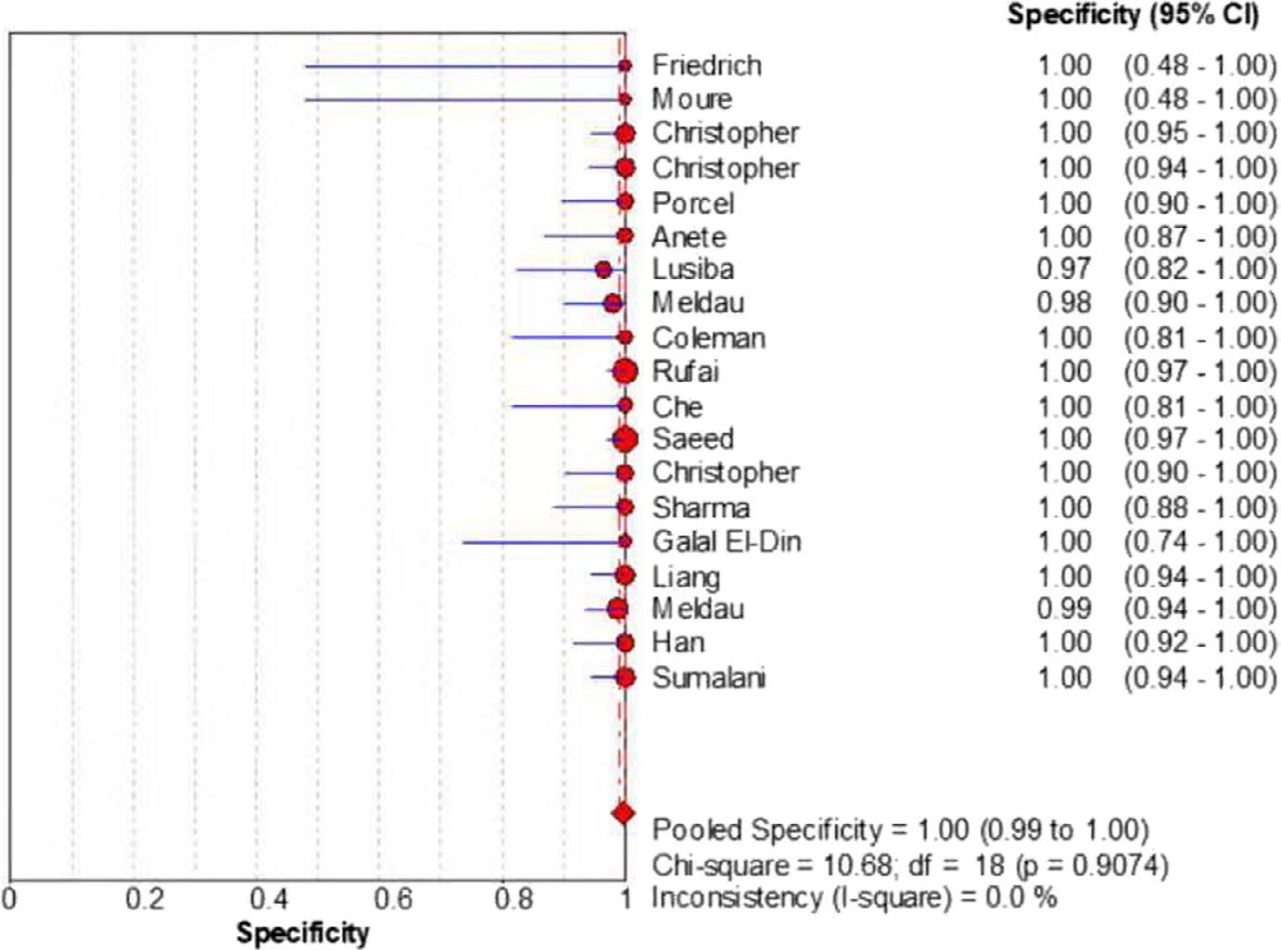
Forest plot of the specificity of the included studies

**FIGURE 5:**
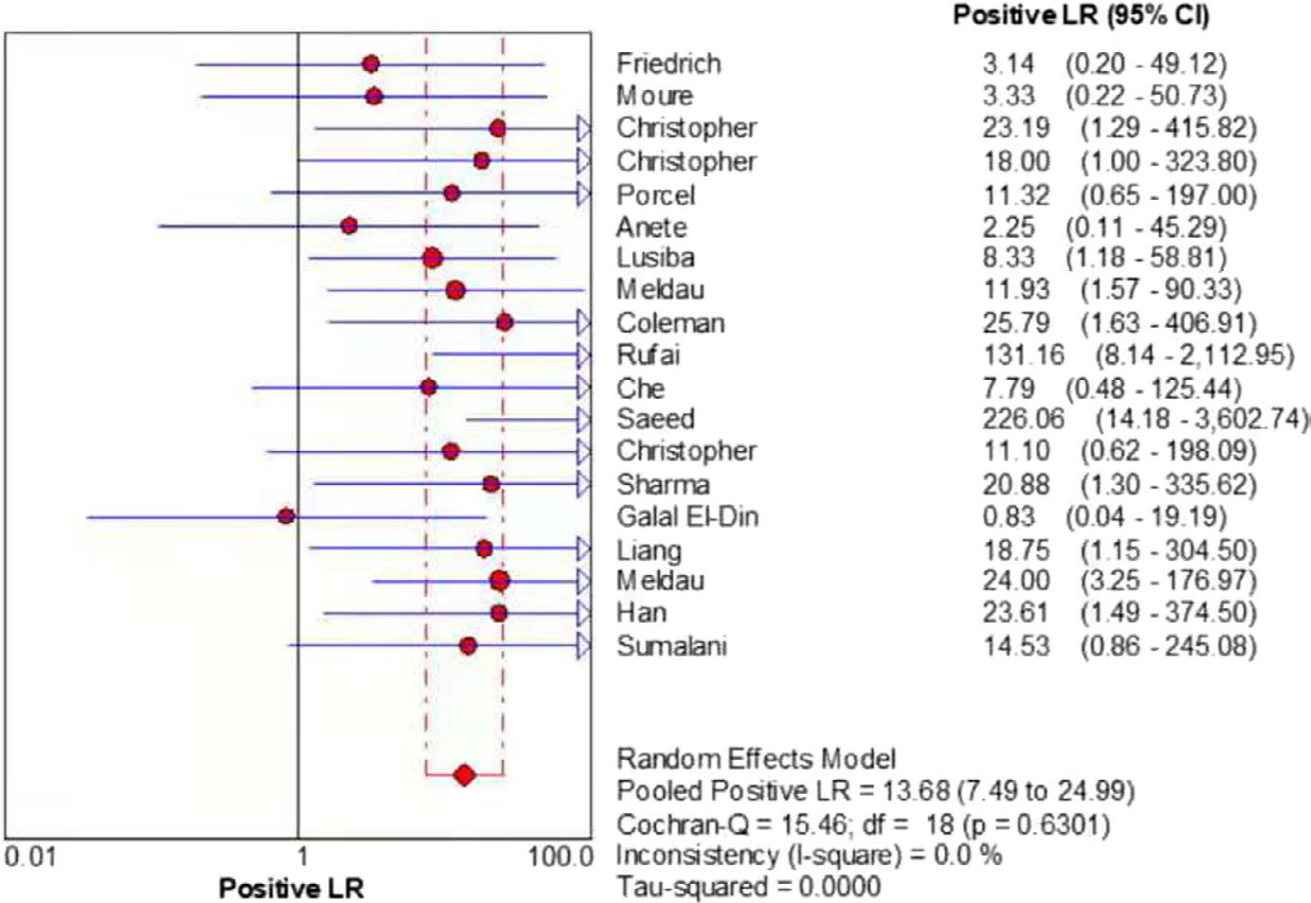
Forest plot of the positive LR of the included studies

**FIGURE 6:**
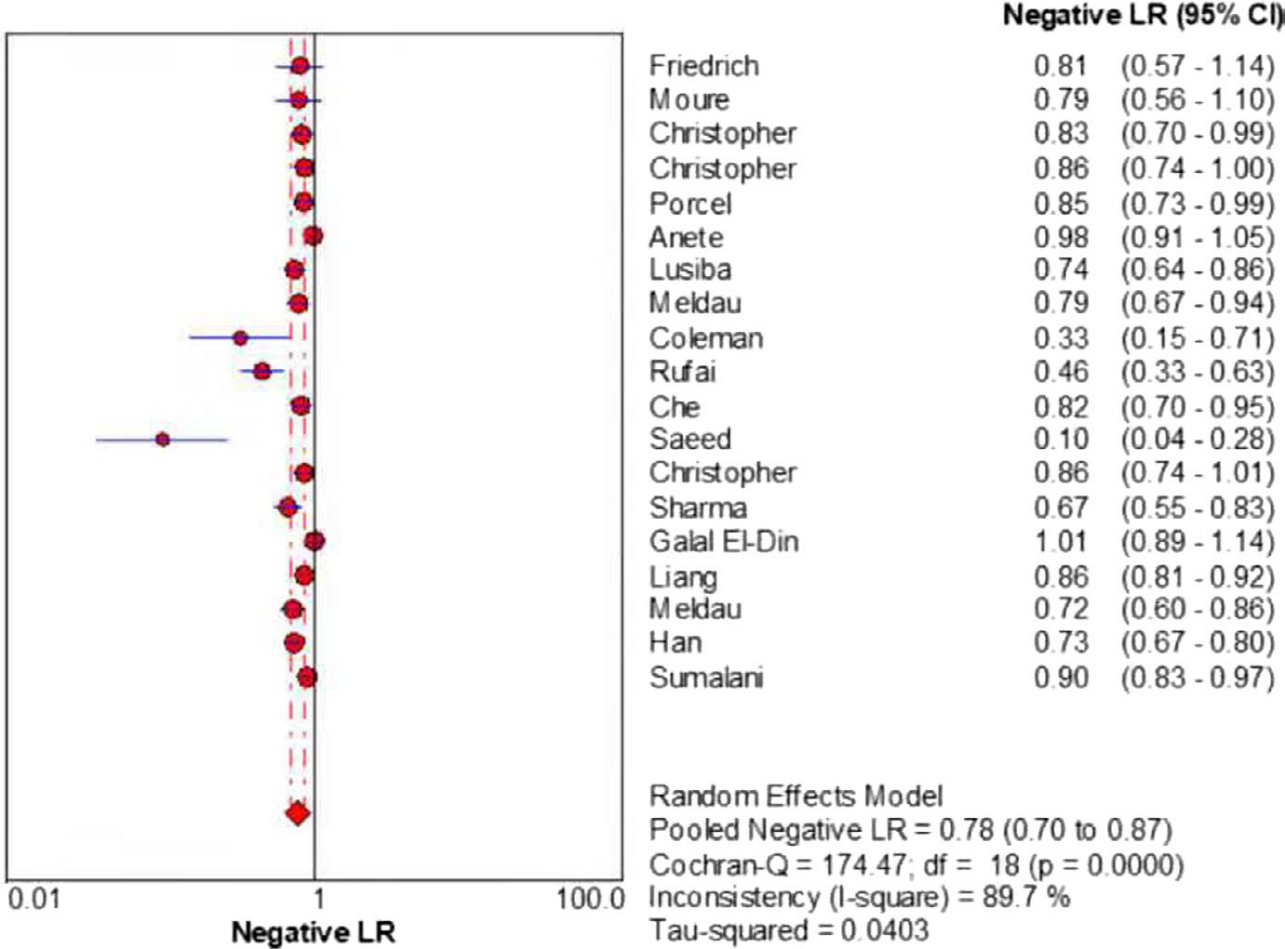
Forest plot of the negative LR of the included studies

**FIGURE 7:**
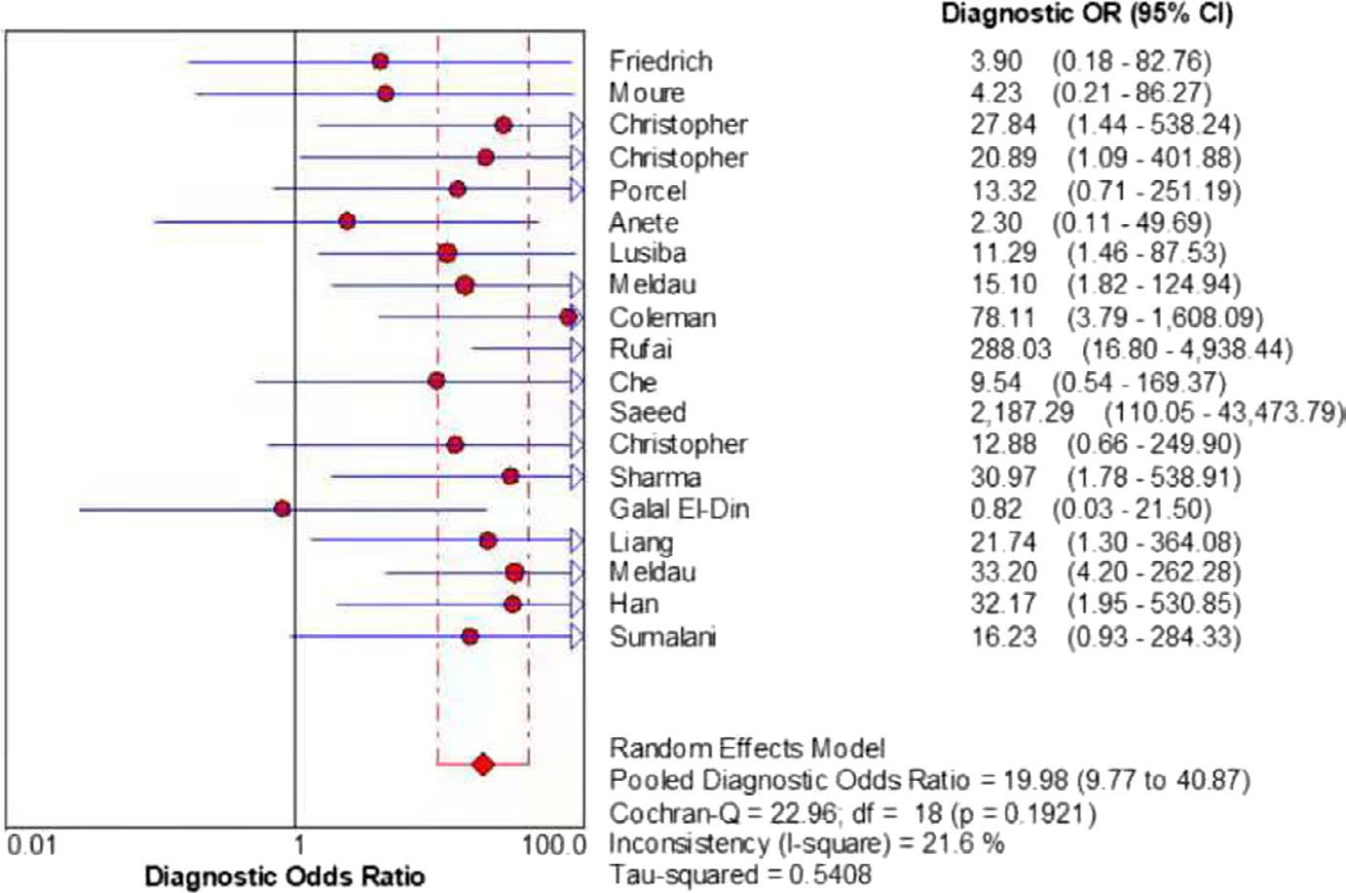
Forest plot of the diagnostic OR of the included studies

**FIGURE 8:**
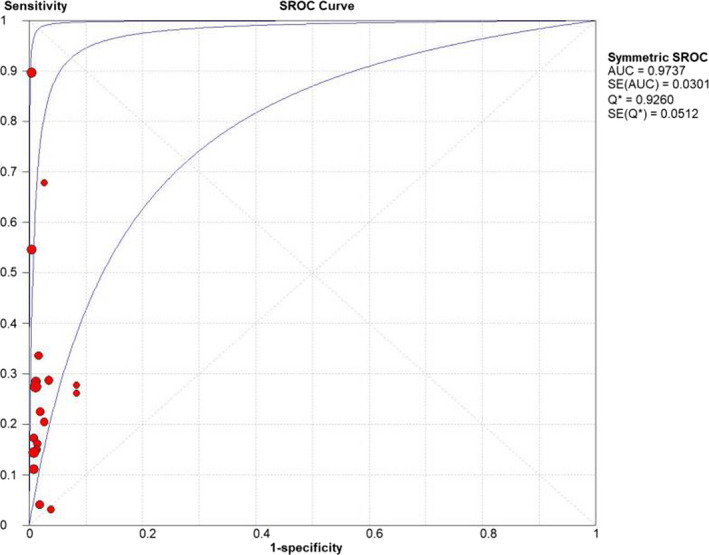
SROC curve of Xpert MTB/RIF for diagnosis of Mycobacterium tuberculosis in tuberculous pleural effusion

The merged sensitivity, specificity, positive LR, negative LR, diagnostic odds ratio, inconsistency (*I*‐square) of DOR values, and area under curve (AUC) of SROC were 0.24 (95% CI: 0.21 to 0.26), 1.00 (95% CI: 0.99 to 1.00), 13.68 (95% CI: 7.49 to 24.99), 0.78 (95% CI: 0.70 to 0.87), 19.98(95% CI: 9.77 to 40.87), 21.6%(<50%), and 0.9737, respectively (Figures 3, 4, 5, 6, 7, 8).

### 3.5 Publication bias

**FIGURE 9:**
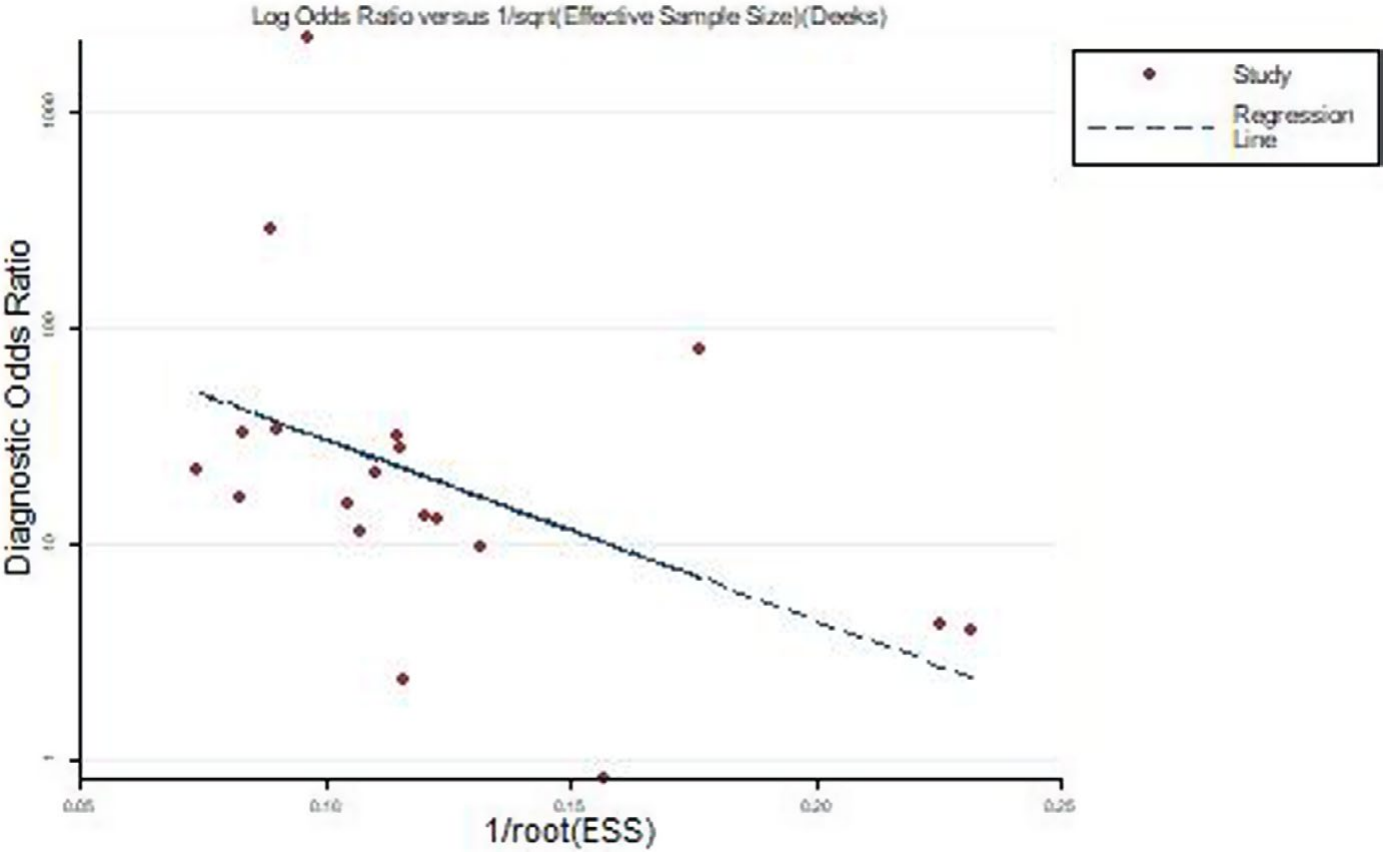
Deek's funnel plot asymmetry test to evaluate publication bias of the included studies for Xpert MTB/RIF detection of tuberculous pleural effusion

In a meta‐analysis, the Deeks funnel plot (Figure 9) generated by Stata 12.0 was used to test the data. The Egger test showed that the *p*‐value of this study was 0.148 > 0.050, indicating that no publication bias was found in the study.

## 4 DISCUSSION

According to the data from World Health Organization in 2019, tuberculosis (TB) remains the leading cause of morbidity and death worldwide, with an annual number of deaths of over 1.4 million, especially in underdeveloped regions.^1^ The conventional laboratory diagnosis of tuberculosis usually adopts Mycobacterium tuberculosis culture, which is time‐consuming, observer‐dependent, and has a low detection rate,^29^, ^30^ leading to certain limitations. In contrast, as a lower cost and faster diagnostic method to detect Mycobacterium tuberculosis in the laboratory, Xpert MTB/RIF greatly improves the efficiency of clinical TB diagnosis. It is recognized by the WHO as significant progress in global TB control and treatment.^31^ Therefore, Xpert MTB/RIF, a rapid diagnostic method of Mycobacterium tuberculosis infection, is of great necessity and importance for patients with corresponding clinical symptoms.

Through the comprehensive search and rigorous filtering of relevant literature, 18 studies were included for meta‐analysis of the diagnosis of Tuberculosis pleural effusion by Xpert MTB/RIF. These 18 articles encompassed 1902 clinical specimens. Results showed that Xpert MTB/RIF had a sensitivity of 0.24 (95% CI: 0.21 to 0.26), a specificity of 1.00 (95% CI: 0.99 to 1.00), a positive LR of 13.68 (95% CI: 7.49 to 24.99), a negative LR of 0.78 (95% CI: 0.70 to 0.87), and a diagnostic odds ratio of 19.98 (95% CI: 9.77 to 40.87). Meanwhile, the SROC curve was plotted, and the following parameters were obtained: AUC of 0.9737 and Q of 0.9260 (SE = 0.0512). The SROC turn was near the top left corner, and the AUC was close to 1, which suggested that Xpert MTB/RIF had a comparatively overall high diagnostic accuracy for pleural effusion. Besides, the inconsistency (*I*‐square) of DOR was 21.6% (<50%), which indicated that there was no heterogeneity.

With the data gained, Xpert MTB/RIF in this study suggested high specificity and low possibility of misdiagnosis. However, its sensitivity was not high enough as a diagnostic method. We suggested that Xpert might be used in combination with other diagnostic methods.

However, the current research still had some limitations. Firstly, we only retrieved and extracted data from the literature published in the four English databases, leading to a lack of comprehensiveness and bias. Secondly, our study only included articles from the beginning of the study through January 2021. In addition, we did not delve into the effects of other potential factors on the results. Finally, the reference standards in each literature were not wholly consistent, making the results have a certain probability of bias.

According to the data analysis of our study, we learned that the low sensitivity of Xpert might be caused by the low bacteria load of mycobacterium in the tuberculous pleural fluid.^15^ Meanwhile, the limited sensitivity probably reflected the presence of inhibitory substances.^14^ Another possible explanation was that the studies included in the analysis used different reference standards.^32^

Although Xpert MTB/RIF for tuberculous pleural effusion was found to be a method with less sensitivity that fails to meet the clinical requirements, its high specificity (100%) suggests it is a specific tool for diagnosis of tuberculous pleural effusion. If the MTB/RIF system result is positive, it indicates Mycobacterium tuberculosis in the pleural effusion. The operation of this technique is simpler than conventional laboratory diagnostic methods. For pleural tuberculosis with a large sample size but low diagnostic rate and microscopic examination positive rate, the technique can still be a method to improve the positive rate of tuberculosis diagnosis. When it is applied in the clinical diagnosis of the disease, in combination with other detection methods such as LAM and culture,^25^ it seemed to improve the sensitivity of the diagnosis of pleural tuberculosis.

## 5 CONCLUSIONS

In summary, our meta‐analysis demonstrated that Xpert MTB/RIF is a rapid and specific diagnostic method for detecting Mycobacterium tuberculosis in pleural effusion with a high specificity of 100%, which can significantly avoid possible misdiagnosis. However, due to its relatively low sensitivity, it is better to be used in combination with other sensitive detection methods if Xpert is required for clinical detection; however, further studies are warranted to confirm these results.

## CONFLICT OF INTEREST

The authors declare that they have no conflicts of interest.

## AUTHORS CONTRIBUTIONS

YR Qiu, YY Chen, XR Wu, YP Li, M Lin, XJ Cao, ZY Yu, QY Li, JC Chen and XG Guo participated in the design of the project, formulation of the search strategy, and determination of inclusion of exclusion criteria. YR Qiu, YY Chen, XR Wu, and YP Li participated in the literature search, data extraction and processing, and quality evaluation. XG Guo has made substantial contributions to the conception and design of the work. YR Qiu, YY Chen, and XR Wu created the figures and table and wrote the manuscript. All researchers read and approved the final version of the manuscript.

## Supporting information

Figure S1
