# Supplementary figures and images for: Accuracy of Xpert MTB/RIF assay for the diagnosis of tuberculous pleural effusion
Source: J Clin Lab Anal. 2021 Dec 17;36(1):e24185. doi: 10.1002/jcla.24185 (PMC8761404; doi:10.1002/jcla.24185)

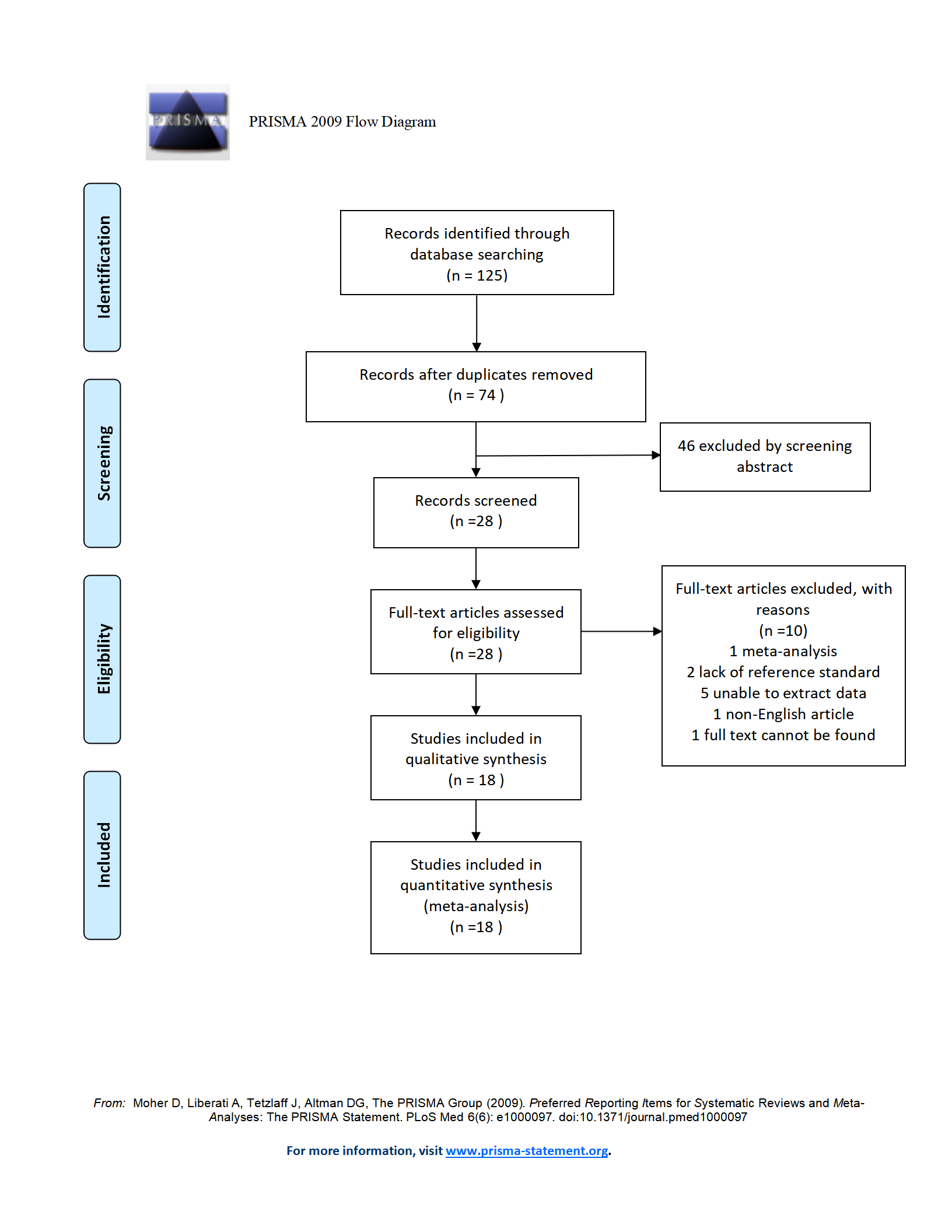

Supplement: Supplementary file 1 — Figure S1 [file JCLA-36-e24185-s001.png]
